# Attitudes of Costa Rican individuals towards donation of personal genetic data for research

**DOI:** 10.2217/pme-2020-0113

**Published:** 2021-02-12

**Authors:** Gabriela Chavarria-Soley, Fernanda Francis-Cartin, Fabiola Jimenez-Gonzalez, Alejandro Ávila-Aguirre, Maria Jose Castro-Gomez, Lauren Robarts, Anna Middleton, Henriette Raventós

**Affiliations:** 1 Escuela de Biología/Universidad de Costa Rica/San José, Costa Rica; 2 Centro de Investigación en Biología Celular y Molecular/Universidad de Costa Rica/San José, Costa Rica; 3 Society & Ethics Research Group, Connecting Science, Wellcome Genome Campus, Cambridge, UK; 4 Faculty of Education, University of Cambridge

**Keywords:** attitudes, data sharing, donation, genomics, survey

## Abstract

**Aim:**

We explore attitudes from the public in Costa Rica regarding willingness to donate DNA data for research.

**Materials & methods:**

A total of 224 Costa Rican individuals answered the anonymous online survey ‘Your DNA, Your Say’. It covers attitudes toward DNA and medical data donation, trust in research professionals and concerns about consequences of reidentification.

**Results & conclusion:**

Most individuals (89%) are willing to donate their information for research purposes. When confronted with different potential uses of their data, participants are significantly less likely to donate data to for-profit researchers (34% willingness to donate). The most frequently cited concerns regarding donation of genetic data relate to possible discrimination by health/life insurance companies and employers. For the participants in the survey, the most trusted professionals are their own medical doctor and nonprofit researchers from their country. This is the first study regarding attitudes toward genetic data donation in Costa Rica.

## Lay abstract

Our study’s aim was to explore attitudes from people in Costa Rica regarding their willingness to donate their own genetic data. A total of 224 Costa Ricans answered an anonymous survey on this topic, as well as on trust in researchers and concerns about data donation. We found that most persons are willing to donate their medical and genetic information for research purposes. However, willingness to donate is not the same for all potential uses of the data. People are more willing to give their information to their medical doctors or for nonprofit research than they are in the case of for-profit research. The most frequently cited concerns regarding donation of genetic data relate to possible discrimination by health/life insurance companies and employers. This is the first study regarding attitudes toward genetic data donation in Costa Rica.

Many research efforts require large amounts of genomic and phenotypic data. In recent years, technological advances and reducing costs of whole exome and whole genome sequencing have resulted in the generation of large amounts of genetic and genomic data worldwide. Some of the contexts in which these data are generated are research projects, biobanks, clinical settings, genetic testing for specific conditions and direct-to-consumer testing. The concept of sharing genomic and phenotypic data within the research community is attractive because it facilitates reaching the large sample size often required in genetic studies and optimizes the use of valuable existing genetic and genomic information. Funding agencies worldwide have developed policies that require data sharing in funded projects [1–3].

While data sharing has many potential benefits, it raises concerns about the privacy of the study participants, including the safeguarding of potentially sensitive health data [4]. By virtue of its scale, the ability to sequence a whole genome to answer questions about health and disease means that there are billions of DNA data points attributable to a research participant; while on their own it may be difficult to connect these to an individual, as a whole (and when considered alongside other personal information available online) it may be possible to reidentify the person whose sample is being researched. This therefore challenges the concept of ‘anonymous’ data donation, and potential identifiability of participants has become a sensitive issue [5,6]. When genomic data are shared, the absolute confidentiality promised in earlier consent forms no longer applies, and this should be made clear during the process of informed consent moving forward [4,7–11]. Even when data are considered anonymous in one database, linking the information with other databases may allow identification of research participants [4–6,12–15]. Some concerns that have been repeatedly raised by research participants relate to discrimination by insurance companies or employers, social stigmatization, and genomic data being used for reidentification for forensic/criminal purposes [15–21].

When a person agrees to share their genetic or genomic information, there are many levels in the scope of future uses of such data: national versus international, academic versus other interests, nonprofit versus for-profit, limited to the phenotype of interest in the study the participant entered versus open to other phenotypes and so on. This casts a spotlight on every detail of the process of informed consent (broad vs narrow, one-time vs reconsenting, etc.) [22–26]. People’s attitudes toward genetic data donation or sharing will influence the extent of what they are willing to consent to. Several factors, such as ethnicity, education, religiosity and levels of perceived benefit and concern [11,19,27–29], have been shown to influence a person’s decision whether to donate their genetic information for research purposes.

We analyze the attitudes of an online sample of Costa Rican individuals toward the donation of genetic data for research purposes. Costa Rica has a decades-old tradition of human genetics research, focusing on both Mendelian and complex disorders [30]. Our research team in particular has over 25 years of experience in neuropsychiatric genetic research in Costa Rica [31]. The population has been very willing to participate in our studies, with over 90% acceptance. Additionally, the vast majority of the participants have agreed to be recontacted throughout the years for follow-up studies and also to participate in meetings in which we return the findings from our studies. The present study gives us the opportunity to determine whether this support exists in the Costa Rican population at a more general level. The data were obtained from the Spanish version of the online survey ‘Your DNA, Your Say’, an international project that has been translated into more than 15 languages [32,33]. In this survey, ‘data donation’ refers to the decision an individual makes to contribute their genomic data to a database that can be accessed by researchers or clinicians. The project’s goal is to collect information on the attitudes regarding donation of genetic data from as many countries as possible, followed by a between-country analysis of attitudes. Different countries are expected to differ in the details of their attitudes toward donation, levels of trust in research professionals, and concerns. We will focus exclusively on the data collected for 224 Costa Rican individuals. To the best of our knowledge, this is the first time that attitudes regarding donation of genetic data have been studied in the country.

## Materials & methods

### Access to survey data

A collaboration was established between H Raventós from the psychiatric genetics research group at the Cell and Molecular Biology Research Center, University of Costa Rica and the researchers who developed the ‘Your DNA, Your Say’ survey from the Wellcome Genome Campus in Cambridge. The survey was developed as part of a collaboration between the Wellcome Genome Campus and the Global Alliance for Genomics and Health, and has been translated into 15 languages. The original English survey for the Your DNA, Your Say project was designed by fluent speakers of English, German, French, Polish, Spanish, Italian and Swedish. The reason for this was so that English words, tone and phrases were chosen (and explained in more detail in the glossary) that were considered easiest to translate into these other languages. Face validity testing was done with experts in genomic data sharing and public engagement, as well as members of the public, to check that the questions made sense in English. When the English survey was translated into other languages, native speakers were used for both the translation and back-translation. The native speakers had expertise in genomics, data sharing and all the technical terms (i.e., they were able to offer face and construct validity), and the back translators did not (i.e., they were able to offer sense checking in lay language). The survey can be accessed at www.YourDNAYourSay.org. It consists of 29 questions and takes approximately 15–20 min to complete [33].

A total of 224 Costa Rican individuals completed the Spanish version of the survey. The completed surveys from these participants were made available to our research group for a separate analysis.

### Recruitment of participants

The 224 participants from Costa Rica voluntarily answered an anonymous survey regarding their views on donation of genetic information. Participants were recruited through announcements in social media and mailing lists, and in the Costa Rican authors’ courses at the university. Due to the recruitment strategy, our sample is not representative of the Costa Rican population in terms of gender, education level and age classes, based on data from the 2011 national census for Costa Rica [34].

A summary of the comparison between our present sample and the data from the 2011 national census is presented in Table 1. While in Costa Rica the sex ratio is 51% female to 49% male, in our study 63% of the participants are female. Regarding age distribution, in the present study the ‘30 or less’ category is underrepresented and the ‘51 or more’ is overrepresented. Only the distribution of marital status in our sample is representative of the Costa Rican population. In terms of education level, the frequency of individuals with tertiary education in our study sample (62%) differs strongly from that in the broader population of Costa Rica (20%). Regarding ethnicity, the categories used in the survey are not directly comparable to those in the census. Both the ‘White’ and ‘Hispanic’ categories from the survey (which together represent 89% of our sample; Table 2), are included in the ‘White or mestizo’ category from the national census (which includes 84% of the population), suggesting that our sample does not differ strongly from the general population in terms of ethnicity.

**Table 1.** Demographic characteristics of the ‘Your DNA, Your Say’ sample compared with the Costa Rican national census (2011).

Variable	Categories	YDYS		2011 census		Census total
		n	%	n	%	
Age	30 and under	55	24.6%	1,285,774	45.1%	2,848,603
	31–50	88	39.3%	1,010,419	35.5%	
	Over 51	81	36.2%	552,410	19.4%	
Gender	Female	141	62.9%	1,861,813	51.4%	3,620,938
	Male	80	35.7%	1,759,125	48.6%	
	Missing	3	1.3%	0	0.0%	
Education	Tertiary	138	61.6%	721,727	20.4%	3,546,316
	Secondary	39	17.4%	560,447	15.8%	
	Other	44	19.6%	2,257,341	63.6%	
	Missing	3	1.3%	6801	0.2%	
Ethnic	Hispanic	168	75.0%	3,597,847	83.6%	4,301,712
	Non-Hispanic	46	20.5%	484,084	11.2%	
	Prefer not to say	10	4.5%	219,781	5.1%	
Relationship	Divorced/single/widowed	117	52.2%	1,720,234	49.6%	3,466,654
	Married/civil partnership/living together	107	47.8%	1,746,420	50.4%	

YDYS: Your DNA, Your Say.

**Table 2.** Participants willing, unwilling and unsure about donating DNA and/or medical information for research purposes (to medical doctors, nonprofit and for-profit researchers) categorized by familiarity with genetics and demographic variables.

Variable	Categories	Unsure (n = 19)		Unwilling (n = 6)		Willing (n = 199)		Total (n = 224)	
		n	%	n	%	n	%	n	%
Genetics knowledge	Unfamiliar	7	36.8%	1	16.7%	48	24.1%	56	25.0%
	Familiar	12	63.2%	5	83.3%	151	75.9%	168	75.0%
Age	30 and under	3	15.8%	2	33.3%	50	25.1%	55	24.6%
	31–50	9	47.4%	1	16.7%	78	39.2%	88	39.3%
	Over 51	7	36.8%	3	50.0%	71	35.7%	81	36.2%
Gender	Female	15	78.9%	3	50.0%	123	61.8%	141	62.9%
	Male	3	15.8%	3	50.0%	74	37.2%	80	35.7%
	Missing	1	5.3%	0	0.0%	2	1.0%	3	1.3%
Children	Yes	8	42.1%	4	66.7%	74	37.2%	86	38.4%
	No	9	47.4%	2	33.3%	121	60.8%	132	58.9%
	Missing	2	10.5%	0	0.0%	4	2.0%	6	2.7%
Education	Secondary	2	10.5%	1	16.7%	36	18.1%	39	17.4%
	Tertiary	12	63.2%	4	66.7%	122	61.3%	138	61.6%
	Other	5	26.3%	1	16.7%	38	19.1%	44	19.6%
	Missing	0	0.0%	0	0.0%	3	1.5%	3	1.3%
Ethnic	Afro-European, African American, Black	0	0.0%	1	16.7%	3	1.5%	4	1.8%
	Hispanic	12	63.2%	2	33.3%	154	77.4%	168	75.0%
	South Asian Indian, Pakistani	0	0.0%	0	0.0%	1	0.5%	1	0.4%
	White	2	10.5%	2	33.3%	28	14.1%	32	14.3%
	Other	2	10.5%	0	0.0%	7	3.5%	9	4.0%
	Prefer not to say	3	15.8%	1	16.7%	6	3.0%	10	4.5%
Religion	A religious person	7	36.8%	1	16.7%	80	40.2%	88	39.3%
	Not a religious person	12	63.2%	5	83.3%	119	59.8%	136	60.7%
Relationship	Divorced/single/widowed	7	36.8%	4	66.7%	106	53.3%	117	52.2%
	Married/civil partnership/living together	12	63.2%	2	33.3%	93	46.7%	107	47.8%

The Your DNA, Your Say project complies with appropriate research ethics policy and data protection regulation governing work at the Wellcome Genome Campus in Cambridge. All surveys were anonymous in that no personally identifiable data (including no IP addresses) were gathered. Consent was deemed implicit if research participants started to answer the survey; they could refuse to complete it and withdraw at any time.

### The survey

For a complete description of the survey, see Middleton *et al.* [33]. Here we briefly present the sections of the survey that were included in our analysis.

Sociodemographic data. Age was registered in 10-year categories, starting at 16. Because of the low number of participants in the younger and older age groups, participants were regrouped into three categories for further analyses: ‘30 years of age or less’, ‘31–50’ and ‘50 or older’. Participants were also asked whether they had children, as well as their relationship status (‘Divorced’, ‘Separated’, ‘Single’, ‘Widowed’, ‘Married/civil partnership/living together’). Participants were asked to self-report their ethnic origin (‘White’, ‘Afro-European/African American, Black’, ‘Hispanic’, ‘South Asian’, ‘Indian’, ‘Pakistani’, ‘East Asian Chinese’, ‘Japanese’, ‘Arabic’, ‘Central Asian’, or ‘Other’). The self-reported highest level of education was categorized as ‘Tertiary’, ‘Secondary’, ‘Primary’ or ‘Other’. Religiosity was self-reported as ‘A religious person’ or ‘Not a religious person’.

Participants were asked whether they had knowledge about genetics (due to personal interest, work or medical/family history) or were unfamiliar with the subject. Throughout the survey, participants answered whether they would donate their ‘anonymous’ DNA and medical information for use by others in research (and in a glossary definition of ‘anonymous’, we explained that the concept of complete anonymity is fallible). They were given a list of scenarios, hypothetical problems or potential concerns that could arise from donating their genetic information. They were asked to choose the three scenarios that concerned them the most. According to Middleton *et al.* [33], the list of hypothetical concerns was generated from pilot work, academic literature and the experience of the Global Alliance of Genomics and Health experts who designed the survey. The list of hypothetical harms presented to participants was:

■ ‘My friends potentially knowing something about me that I hadn’t chosen to tell them’;
■ ‘My family potentially knowing something about me that I hadn’t chosen to tell them’;
■ ‘My government potentially knowing something about me that I hadn’t chosen to tell them’;
■ ‘Police potentially knowing something about me that I hadn’t chosen to tell them’;
■ ‘Marketing companies targeting me to sell me products’;
■ ‘Being stigmatized and labeled in some way online’;
■ ‘Being cloned’;
■ ‘My DNA being copied and then planted at the scene of a crime’;
■ ‘Health or life insurance companies using the information to discriminate against me’;
■ ‘Employers using the information to discriminate against me’;
■ ‘Upsetting my genetic relatives’;
■ ‘Ethnic identification and racial discrimination’.

Participants were also asked whether they would trust the following to use their medical or genetic information:

■ ‘My medical doctor’;
■ ‘Any medical doctor in my country’;
■ ‘Any medical doctor worldwide’;
■ ‘Any researcher at a university in my country’;
■ ‘Any researcher at a university worldwide’;
■ ‘Any researcher at a company in my country’;
■ ‘Any researcher at a company worldwide’;
■ ‘The government of my country’;
■ ‘Governments worldwide’.

They had to choose one of three possible answers: ‘I would generally trust’, ‘I would generally not trust’, or ‘I'm just not sure’.

### Willingness to donate DNA & medical information

The participants' willingness to donate their DNA and medical information was classified in the same way as the previous analysis for the English-speaking countries [19]. Throughout the survey, participants were asked whether they would donate their ‘anonymous’ DNA and medical information for use in research. Participants indicated who they would allow to use their data, out of three categories: medical doctors, nonprofit researchers or for-profit researchers. Participants were classified as ‘willing to donate’ if they answered ‘yes’ to at least one of these (showing that they were not opposed to medical and genetic data donation *per se*). Participants who answered ‘no’ to all three were classified as ‘unwilling to donate’, and those who answered ‘unsure’ to all three, or had a combination of ‘unsure’ and ‘no’ answers were classified as ‘unsure’.

### Statistical analysis

Sample characteristics regarding familiarity with genetics and sociodemographic aspects are summarized. A multivariable analysis of participant characteristics associated with donation preference (willing, unwilling, unsure) was performed using the «nnet» R package [35] only in the case of for-profit research, using a multinomial logistic regression model with donation preference as the outcome variable. Age, gender, ethnicity, country of residence, relationship status, having children, education level, religiosity and familiarity with genetics were included as covariates. For the other two categories of potential users of genetic data (medical doctors and nonprofit researchers), this multivariable statistical analysis could not be performed due to the very small number of individuals unwilling to donate their information.

## Results

A total of 224 Costa Rican individuals voluntarily answered the Your DNA, Your Say online survey. When participants were asked whether they would donate their ‘anonymous’ DNA and medical information for different categories of research (medical doctors, nonprofit researchers, for-profit researchers) 6 out of 224 participants answered ‘no’ to all categories and were therefore classified as ‘unwilling to donate’. Another 19 individuals were classified as ‘unsure’ because either they answered ‘unsure’ to all three categories of research or their answers were a combination of ‘unsure’ and ‘no’. A total of 199 individuals, representing 89% of the sample, were willing to donate their DNA and medical information to at least one kind of research professional. The characteristics of the sample and the willingness of individuals in the different categories to donate their genetic information for research purposes are summarized in Table 2.

**Figure 1.**
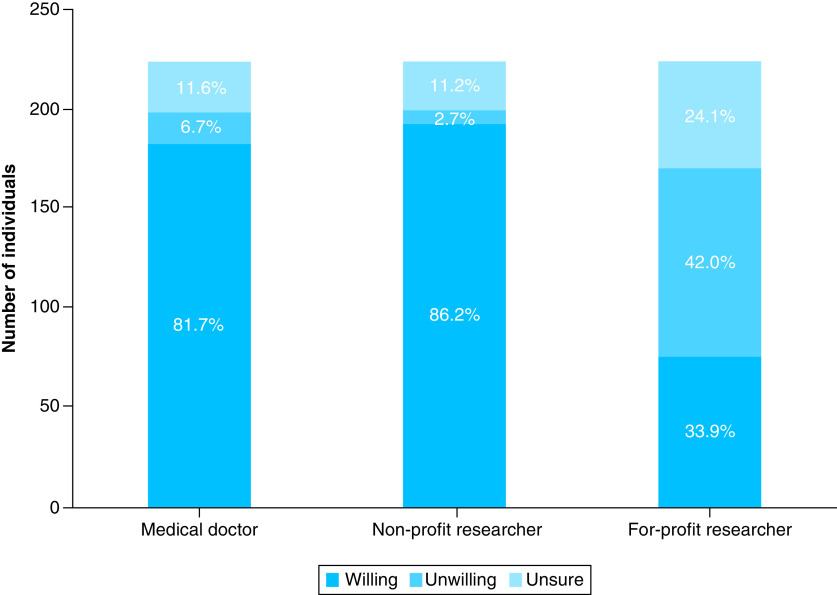
Distribution of willingness to donate genetic information for different research interests or categories.

We found that willingness to donate differed significantly according to the potential use of the data (Figure 1). While most participants were willing to donate information to medical doctors and nonprofit researchers, willingness to donate decreased strongly in the case of for-profit research (X^2^ = 193.6; degrees of freedom [df] = 4; p < 0.0001).

For statistical reasons, given the very small number of individuals who were either unwilling or unsure regarding donation of their genetic data to medical doctors and nonprofit researchers, a multinomial regression for willingness to donate according to familiarity with genetics and demographic data could only be performed for the for-profit research category. For this category, when comparing people who were either willing to donate or unsure about donating versus the group of people who were unwilling to donate, no significant differences regarding familiarity with genetics or sociodemographic factors could be found (Table 3).

**Table 3.** Multinomial logistic regression result for views on donation to for-profit researchers, with ‘unwilling to donate’ as reference category, associated with familiarity about genetics and demographic data.

Variable	Category	Willing				Unsure			
		OR	LCI	UCI	p-value	OR	LCI	UCI	p-value
Familiar with genetics	Familiar	ref				ref			
	Unfamiliar	1.07	0.50	2.28	0.43	1.56	0.69	3.55	0.14
Age	31–50	ref				ref			
	Over 51	0.75	0.34	1.65	0.24	0.80	0.33	1.94	0.26
	30 and under	1.39	0.55	3.49	0.24	1.40	0.50	4.00	0.31
Gender	Female	ref				ref			
	Male	0.62	0.32	1.22	0.08	0.58	0.27	1.25	0.08
Children	No	ref				ref			
	Yes	1.36	0.62	2.97	0.22	1.18	0.49	2.85	0.35
Tertiary education	Yes	ref				ref			
	No	1.08	0.56	2.11	0.40	1.24	0.58	2.62	0.29
Ethnicity	Hispanic	ref				ref			
	Non-Hispanic	1.03	0.49	2.14	0.47	0.53	0.21	1.34	0.09
Religious person	No	ref				ref			
	Yes	1.49	0.77	2.87	0.11	1.85	0.89	3.82	0.05
Relationship	Divorced/single/widowed	ref				ref			
	Married/civil partnership/living together	0.90	0.45	1.83	0.39	1.07	0.48	2.39	0.43

LCI: Lower 95% confidence interval; OR: Odds ratio; ref: Reference; UCI: Upper 95% confidence interval.

**Figure 2.**
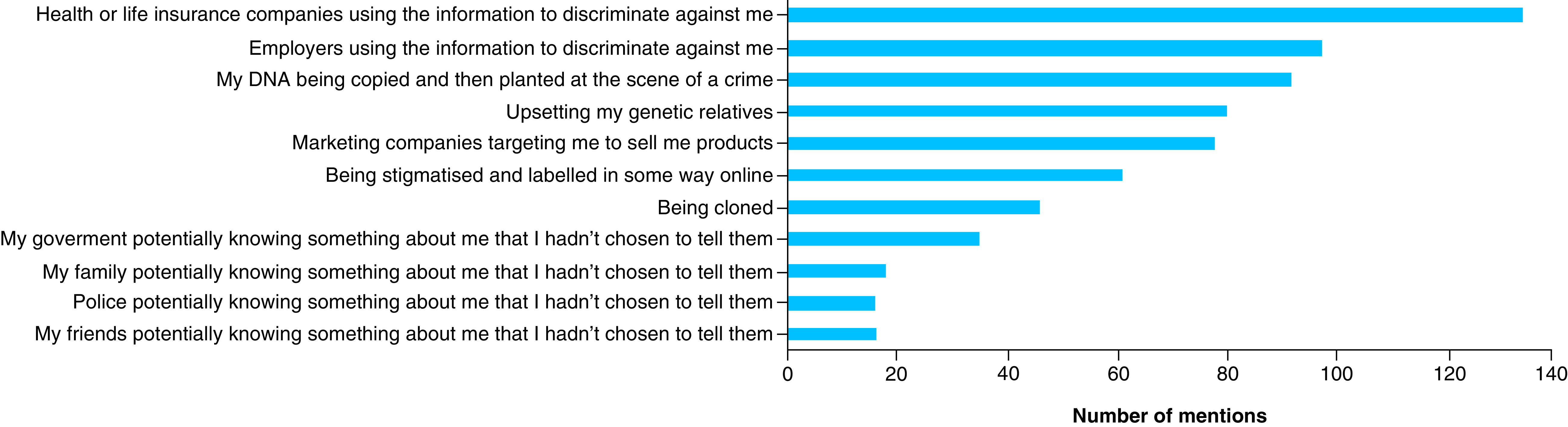
Top concerns regarding donation of medical and DNA information.

In the survey, participants were asked to name their top three concerns regarding the donation of their medical or DNA information, out of a list of potential concerns. As can be seen in Figure 2, the highest concern was ‘Health or life insurance companies using the information to discriminate against me’ (mentioned 133 times). This was followed by ‘Employers using the information to discriminate against me’ and ‘My DNA being copied and then planted at the scene of a crime’ (mentioned 97 and 92 times, respectively).

**Figure 3.**
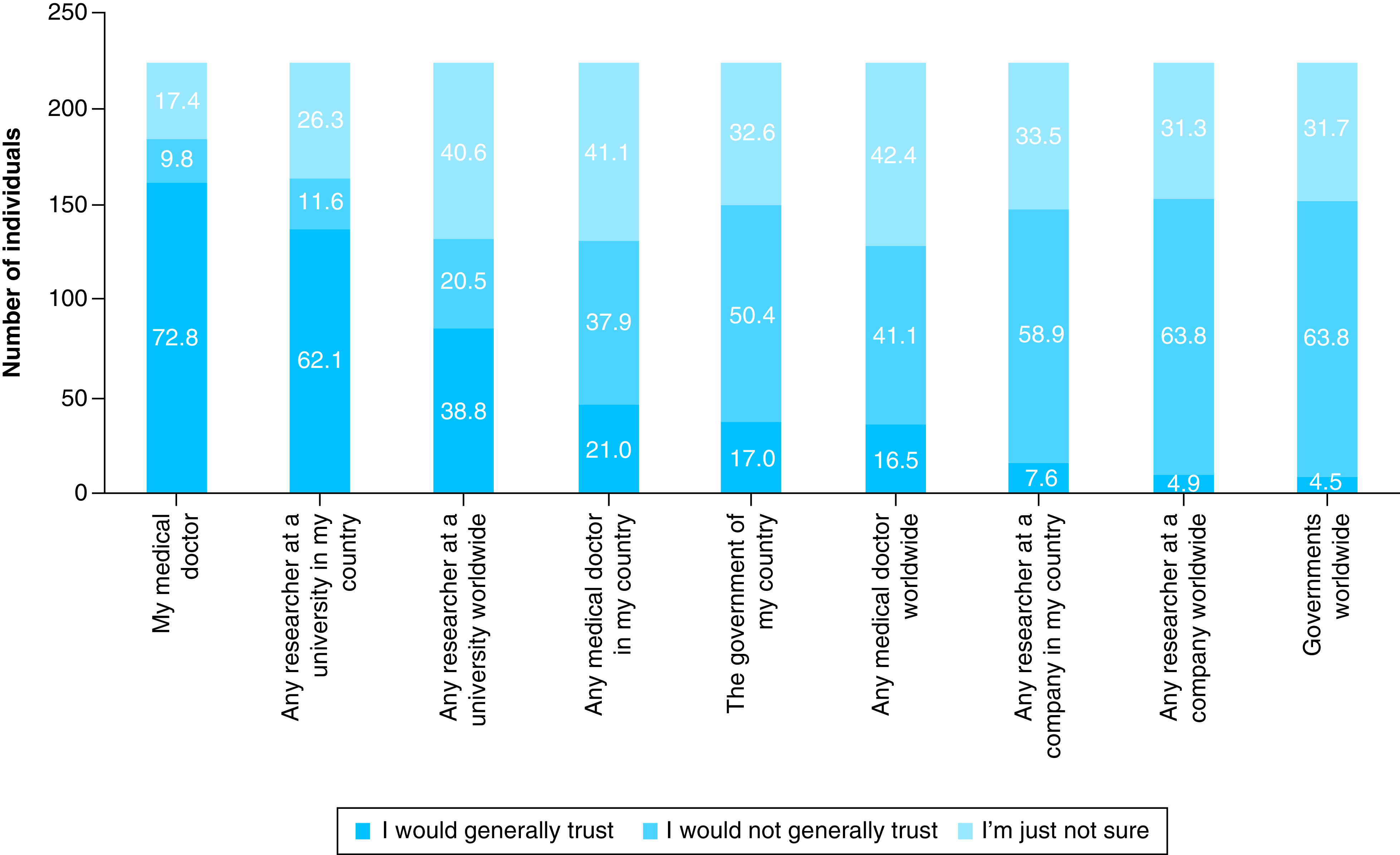
Level of trust in different professionals regarding the use of medical and genetic information. Percentage values for the trust categories are shown in the bars.

The survey also inquired whether participants would trust different kinds of professionals and governments with their information (Figure 3). The level of trust differed significantly between categories, with the person’s own medical doctor and researchers at a university in the participant’s country presenting the highest levels of trust (X^2^ = 660.9; df = 16; p = 2.6E-130).

## Discussion & conclusion

The ‘Your DNA, Your Say’ study is an international program of research that uses an online cross-sectional survey to gather global public attitudes toward the donation, access and sharing of DNA information [32,33]. The first reports with results from the study have included data based on answers from English-speaking individuals from the UK, the USA, Canada and Australia [19,36]. Here we present the results for the Costa Rican sample of individuals who participated in the study.

It is important to emphasize that, due to the way recruitment was performed in our study (see methods), our sample cannot be considered representative of the Costa Rican population. Our sample mainly includes people with university studies and some familiarity with genetics (Table 2). However, in spite of this bias, our results reflect interesting aspects regarding the attitude of this particular set of individuals toward donation of genetic data.

Approximately 89% (n = 199) of the 224 individuals in our sample are willing to donate their medical and genetic information to at least one of the three categories of research included in the survey (medical doctors, nonprofit researchers and for-profit researchers). This is a much higher value than the one in the results of Middleton *et al.* [19], where around 68% of individuals were willing to donate in at least one scenario. Less than 3% (n = 6) of the Costa Rican participants are unwilling to donate their information, and approximately 8.5% (n = 19) are unsure. In the Middleton *et al.* study [19], the ‘unwilling’ and ‘unsure’ categories each included around 16% of the sample. The Middleton *et al.* findings report attitudes from representative public audiences from the UK, the USA, Canada and Australia. It is possible that differences between these results and ours from Costa Rica could be accounted for by the higher education level of our sample. Alternatively, our results could reflect a higher willingness to donate genetic information in Costa Rica when compared with the previously analyzed English-speaking countries. Our own experience with three decades of genetic research projects in Costa Rica is that participation is very high (over 90%). Participants in our studies have mainly been families with a high prevalence of psychiatric disorders [31].

However, a clear and statistically significant difference could be observed in the participants’ willingness to donate according to who would be using their medical or genetic information. While the vast majority of individuals are willing to donate their information to medical doctors and nonprofit researchers (over 80% in both cases), when it comes to for-profit researchers, only 34% of individuals are willing to donate their information. Different studies have found that willingness to donate genetic data to private companies decreases when compared with academic research purposes [36–40]. Individuals usually have altruistic motivations for data sharing, and this is coupled with a negative view of for-profit use or privatization of their information [37,41].

As discussed above, statistical analysis exploring the relationship between willingness to donate and familiarity with genetics and sociodemographic variables could only be performed for the for-profit research use of the data. No significant associations were found, possibly due in part to the small sample size. The assignment of participants to ethnicity categories in self-administered global surveys such as Your DNA, Your Say is a subject that merits some discussion. In our sample, for example, 75% of participants classified themselves as ‘Hispanic’ and 14% as ‘White’. In Costa Rica’s national census, however, these two categories are part of one broad category, ‘White or mestizo’, which includes 84% of the population [34]. This example illustrates a challenge in the design and data analysis of international surveys, where the interpretation of results often requires a careful country-by-country analysis. This issue is especially important because ethnicity has been reported to be one of the factors influencing the willingness to share genetic data [11,28,29]. When the Your DNA, Your Say project was designed, surveys from the World Health Organization were used to guide the structuring of the ethnicity question in the original English version of the survey. The intent for this survey, and the first few translations, was to gather data across Europe. But as interest in translating the survey grew and more countries became involved in the project, it became apparent that the ethnicity question was not sophisticated enough to capture nuance in certain settings. The project team decided to keep the original ethnicity question as it was for consistency. For future research, this question will be expanded considerably to give more options. However, we are mindful that there is no consistently used, validated measure of ethnicity that is routinely used globally, that a global public agrees with.

When participants were asked to choose their three main concerns regarding donation of genetic data from a list, the number one concern was ‘Health or life insurance companies using the information to discriminate against me’. This concern was in second place in the study including survey data from English-speaking individuals in the UK, USA, Canada and Australia [19]. For the Costa Rican sample, ‘Employers using the information to discriminate against me’ was in second place. Genetic discrimination in insurance and employment contexts has been one of the most discussed potential risks surrounding the sharing of genetic and genomic information. Worldwide, different countries have taken varying approaches to address this risk; these include the Genetic Information Nondiscrimination Act in the USA, a voluntary moratorium on the use of genetic data in the UK and formal legal prohibitions in other European countries, to name a few examples [42,43]. In Costa Rica there is no legislation referring to genetic discrimination. It can be argued that the existence of a public universal healthcare system in the country provides individuals with protection against discrimination in the health context. However, this system coexists with an increasingly stronger private health and life insurance system, which probably explains the participants’ concerns. Interestingly, the third concern in our study – and the first concern among the English-speaking survey participants – was ‘My DNA being copied and then planted at the scene of a crime’, which could be a result of exposure to media and popular culture [44–49].

In our sample there is a high level of trust in the person’s own medical doctor and researchers at a university in the person’s own country (Figure 3), which is in agreement with the results on willingness to donate to different categories of researchers (Figure 1). Participants view their own medical doctors and scientists at universities (nonprofit research) as trustworthy. Trust levels in research done by companies and trust in the government are much lower. Costa Rican data from the Wellcome Trust Global Monitor [50] support this view; when asked about trust in different people (neighbors, national government, scientists, journalists, doctors and nurses, people who work at NGOs, traditional healers), the first two most trusted groups in Costa Rica were doctors/nurses and scientists.

The results regarding trust can be compared with the results from the Your DNA, Your Say survey that have been published for the English-speaking countries (UK, USA, Canada, Australia) [36]. Trust in the person’s own medical doctor is very similar between the two studies (73% in Costa Rica vs 75% in the English-speaking countries), and the same is seen for trust in the person’s own government (17% in Costa Rica vs 19% in the English-speaking countries). While both studies show a much lower trust in researchers working for a company in the participant’s country, this effect is stronger in Costa Rica (7.6% in Costa Rica vs 13.3% in the English-speaking countries). Most interestingly, the level of trust in researchers at a university in the participant’s own country is almost double in Costa Rica (62% in Costa Rica vs 34% in the English-speaking countries). The Costa Rican results seem to show a high level of trust in research at universities (usually public universities with mostly public funding of research), coupled with distrust of for-profit research done by companies. This result could also be related to the higher education level in our sample. Public universities in Costa Rica routinely obtain the highest scores in public opinion polls, from a list of several other government institutions [51]. One of the practical implications of our results is that the high level of trust in public research, together with the existence of a unified health system, suggests that the establishment of a national biobank in Costa Rica could be feasible, although significant bureaucratic and regulatory hurdles would need to be overcome.

To the best of our knowledge, this is the first report of the results from the survey Your DNA, Your Say in a Central American country. Because our participation in the survey was small and biased toward participants with higher education, future studies should be conducted with a representative Costa Rican sample to determine if our findings are replicated. We would also like to elucidate whether the differences observed between our results and North American and European countries are also seen in other Latin American countries. Given that attitudes toward donation of genetic information and trust in research can potentially be influenced by many factors, it will be interesting to study the geographical variations. The Your DNA, Your Say survey is a valuable tool that will allow such comparisons and analyses.

**Executive summary**

- We explored the attitudes of 224 Costa Rican individuals regarding donation of their DNA and medical data for research purposes.
- Participants answered the ‘Your DNA, Your Say’ anonymous survey in Spanish.
- Your DNA, Your Say is an international project which aims to collect and compare the views of individuals from different countries on sharing of genetic data.
- 89% of the participants are willing to donate their DNA and medical information to at least one kind of research professional (from three categories: medical doctor, nonprofit researcher and for-profit researcher).
- Willingness to donate differs significantly according to the potential user of the data; it is much lower in the case of for-profit researchers as recipients of the data.
- The most frequently cited concerns surrounding data reidentification relate to possible discrimination by health or life insurance companies and employers.
- Participants were most likely to trust their own medical doctor and nonprofit researchers from their own country with their data.
- We found a greater willingness to share genetic information for research than has been reported for other non-Latin American countries, coupled with a high level of trust in nonprofit research and health professionals.
